# Early insights from the Healthy Life Trajectories Initiative

**DOI:** 10.2471/BLT.26.295804

**Published:** 2026-07-27

**Authors:** Michelle Pentecost

**Affiliations:** a Department of Global Health and Social Medicine, Faculty of Social Science and Public Policy, Room 3.19, level 3, Bush House NE Wing, King’s College London, Strand, London WC2R 2LS, England.

The research field of developmental origins of health and disease emerged about four decades ago to assess how exposures in early life affect future health outcomes. While the relationship between early life exposures and future health outcomes was strongly established on the basis of association studies, there have been fewer intervention studies. One of the cornerstones of evaluating early life interventions is the Healthy Life Trajectories Initiative, known as HeLTI, which is an international consortium overseen by the World Health Organization (WHO) and established in 2017. The initiative runs four harmonized intervention trials in Canada,^1^ China,^2^ India^3^ and South Africa^4^ to test whether a four-phase complex intervention, from preconception through pregnancy, infancy and childhood, improves developmental and metabolic outcomes. The results of the initiative will shape the way we approach and understand early life interventions, and provide a landmark in life-course epidemiology and in developmental origins of health and disease research.

The primary outcome of the initiative is whether the interventions have an impact on childhood adiposity outcomes at 5 years of age, and secondary outcomes cover both maternal and child health. The harmonized interventions consist of micronutrient supplementation and behaviour change interventions modelled on Healthy Conversation Skills,^5^ a motivational interviewing technique based on person-centred communication. These interventions are delivered by public health nurses or community health workers to closely mirror public health infrastructures in each country represented in the consortium to demonstrate scalability of the intervention.

The initiative is already transforming how we conceptualize and conduct life-course research, as its design brings organizational, conceptual and methodological innovations. First is its funding structure: the Canadian Institutes of Health Research, the National Natural Science Foundation of China, India’s Department of Biotechnology and the South African Medical Research Council jointly committed to a shared funding model. This transnational funding partnership, supported by WHO as convenor, is unusual and demonstrates investment in building something no single agency could create alone.

Conceptually, the initiative pioneers the systematic testing of preconception as an intervention point within a life-course framework. While trials such as WINGS^6^ have shown that multidomain interventions from preconception through early childhood produce modest improvements in neurodevelopment at 24 months, the initiative is the first to implement a harmonized preconception trial across four diverse contexts. Nearly 2000 variables have been prospectively harmonized across sites, alongside intervention domains, biospecimen collection, analytical protocols and outcome measures. This approach creates statistical power for pooled analyses, enables cross-context comparison and establishes a data platform of exceptional value to the wider scientific community.^7^ Importantly, the harmonization process, spanning three and a half years, also clarified what cannot be harmonized, itself a useful contribution to global trial design.

Methodologically, the initiative breaks new ground by unblinding cohorts after each phase of the four-phase trial. This novel approach to the problem of testing the effects of a complex intervention on outcomes some years into the future makes an important contribution to debates around what constitutes an explanatory or a pragmatic trial.^8^ In 2018 the consortium convened a panel that included experts in developmental origins of health and disease, statistics, epidemiology, clinical trials and ethics, as well as journal editors and research funders to debate the advantages and disadvantages of early unblinding, agreeing on a pragmatic approach to allow linked mechanistic studies (that is, additional studies alongside the main trial to understand how the intervention works) and secondary outcome comparative analyses between groups (that is, comparing the groups on additional results beyond the main outcome).^9^ The initiative thus shifts emphasis from a narrow focus on primary outcomes towards seeing the consortium as a learning platform generating knowledge throughout its lifecycle.

This approach has proven crucial amid the profound global changes since the initiative’s inception. Conceived in the late 2010s, a period marked by optimism about multilateralism, the initiative has persisted through the coronavirus disease 2019 pandemic, major geopolitical upheaval and rapid advances in data science, all of which have shaped global health research. The consortium was designed for a markedly different environment than the one that exists today. The initiative stands as an important exemplar of transnational collaboration among countries, funders and scientists. The relationships underpinning it have required immense trust and have endured several crises that have reshaped the global health landscape.

The initiative’s results to date include an important body of individual trial publications^10^ that have documented baseline data in each cohort as well as process evaluation. As of March 2026, all trials have completed their preconception phase and the consortium can commence comparative secondary analyses of Phase I.

Several consortium-level insights are also becoming clear. First, the initiative demonstrates how a consortium can function as a learning platform. While initially focused on childhood obesity, the initiative now also covers cardiometabolic health, mental health, gender dynamics and more; its biobanks and data sets can be used to address contemporary research questions and provide material for future analyses. 

Second, the initiative illustrates the effort required to truly adapt to context and attend to complexity. Doing so is far more challenging than pursuing simplistic solutions that rarely succeed. What will emerge from the initiative is not a narrow set of context-blind recommendations but principles for designing life-course interventions that are responsive to local realities and prioritize equity across health, gender, disability, family structures and reproductive life plans. 

Third, the initiative foregrounds gender equity in life-course approaches. Teams have contributed meaningfully to debates on how preconception interventions should be designed and framed to protect gender equity.^11^

Finally, the initiative is a model of transnational collaboration for global health; its work showcases what is possible when partners remain committed despite political divergence, financial uncertainty and competing national priorities. In a period marked by isolationism, withdrawal from multilateral commitments and a leadership vacuum in global health, the initiative illustrates the indispensable role of WHO as a convening body that democratizes knowledge production. At a moment when the value of global health governance is increasingly questioned, the initiative shows how multilateral partnerships remain essential to answer questions of global relevance and why governments should continue to fund research that benefits populations worldwide.

The initiative is more than a set of trials: it is a demonstration of how early-life interventions can be conceptualized, tested and implemented across contexts, and how scientific collaboration can be sustained in challenging contexts. However, the model does have weaknesses: it is resource- and time-intensive, and the consortium must continually balance the challenge of running individual trials alongside ensuring a harmonized approach. The initiative offers important lessons beyond specific outcomes for shaping principles and infrastructures in future global health research. The initiative has demonstrated the limits of prospective harmonization, the resource and coordination challenges of a 10-year project and the extent to which such endeavours must weather unforeseen circumstances. 
